# Plasma levels and tissue expression of liver-type fatty acid-binding protein in patients with breast cancer

**DOI:** 10.1186/s12957-023-02944-8

**Published:** 2023-02-18

**Authors:** Chi-Chang Chang, Chia-Chang Hsu, Teng-Hung Yu, Wei-Chin Hung, Shyh-Ming Kuo, Chia-Chi Chen, Cheng-Ching Wu, Fu-Mei Chung, Yau-Jiunn Lee, Ching-Ting Wei

**Affiliations:** 1 Department of Obstetrics & Gynecology, E-Da Hospital/E-Da Dachang Hospital, Kaohsiung, 82445 Taiwan; 2 School of Medicine for International Students, College of Medicine, I-Shou University, Kaohsiung, 82445 Taiwan; 3 Division of Gastroenterology and Hepatology, Department of Internal Medicine, E-Da Hospital, Kaohsiung, 82445 Taiwan; 4 Health Examination Center, E-Da Dachang Hospital, Kaohsiung, 80794 Taiwan; 5 The School of Chinese Medicine for Post Baccalaureate, College of Medicine, I-Shou University, Kaohsiung, 82445 Taiwan; 6 Division of Cardiology, Department of Internal Medicine, E-Da Hospital, Kaohsiung, 82445 Taiwan; 7 School of Medicine, College of Medicine, I-Shou University, Kaohsiung, 82445 Taiwan; 8 Department of Pathology, E-Da Hospital, Kaohsiung, 82445 Taiwan; 9 College of Medicine, I-Shou University, Kaohsiung, 82445 Taiwan; 10 Lee’s Endocrinologic Clinic, Pingtung, 90000 Taiwan; 11 Division of General Surgery, Department of Surgery, E-Da Hospital, No. 1, Yi-Da Rd., Jiau-Shu Village, Yan-Chao Township, Kaohsiung, 82445 Taiwan; 12 Department of Biomedical Engineering, I-Shou University, Kaohsiung, 82445 Taiwan; 13 Department of Electrical Engineering, I-Shou University, Kaohsiung, 82445 Taiwan

**Keywords:** Liver-type fatty acid-binding protein, Concentrations, Expression, Breast cancer

## Abstract

**Background:**

Liver-type fatty acid-binding protein (L-FABP) is widely expressed in hepatocytes and plays a role in lipid metabolism. It has been demonstrated to be overexpressed in different types of cancer; however, few studies have investigated the association between L-FABP and breast cancer. The aim of this study was to assess the association between plasma concentrations of L-FABP in breast cancer patients and the expression of L-FABP in breast cancer tissue.

**Method:**

A total of 196 patients with breast cancer and 57 age-matched control subjects were studied. Plasma L-FABP concentrations were measured using ELISA in both groups. The expression of L-FABP in breast cancer tissue was examined using immunohistochemistry.

**Result:**

The patients had higher plasma L-FABP levels than the controls (7.6 ng/mL (interquartile range 5.2–12.1) vs. 6.3 ng/mL (interquartile range 5.3–8.5), *p* = 0.008). Multiple logistic regression analysis showed an independent association between L-FABP and breast cancer, even after adjusting for known biomarkers. Moreover, the rates of pathologic stage T2+T3+T4, clinical stage III, positive HER-2 receptor status, and negative estrogen receptor status were significantly higher in the patients with an L-FABP level greater than the median. Furthermore, the L-FABP level gradually increased with the increasing stage. In addition, L-FABP was detected in the cytoplasm, nuclear, or both cytoplasm and nuclear of all breast cancer tissue examined, not in the normal tissue.

**Conclusions:**

Plasma L-FABP levels were significantly higher in the patients with breast cancer than in the controls. In addition, L-FABP was expressed in breast cancer tissue, which suggests that L-FABP may be involved in the pathogenesis of breast cancer.

**Supplementary Information:**

The online version contains supplementary material available at 10.1186/s12957-023-02944-8.

## Background

Breast cancer is the most common cancer and the leading cause of cancer deaths in women worldwide [1]. Epidemiological studies have demonstrated that patients who are overweight/obese and have diabetes and metabolic syndrome are at an increased risk of breast cancer [2–5]. Furthermore, increasing evidence has supported an association between nonalcoholic fatty liver disease (NAFLD) and extrahepatic cancers such as breast cancer [6–8]. In addition, patients with breast cancer and NAFLD have also been reported to have a poorer prognosis in terms of recurrence [9]. Moreover, we previously found that fatty acid-binding protein (FABP)-1 may be involved in the pathogenesis of NAFLD in patients with type 2 diabetes mellitus [10].

The FABP families act as intracellular fatty acid transporters. They are involved in lipid metabolism and play a role in regulating cellular metabolism and inflammation through interactions with peroxisome proliferator-activated receptors (PPARs) [11–16]. One member of this family, liver-type fatty acid-binding protein (L-FABP), also known as FABP1, is located at chromosome 2p12-q11 and is highly expressed in hepatocytes, as well as renal tubular cells, enterocytes, and the alveolar epithelium of the lung [17, 18]. Direct interactions between PPARγ and L-FABP have been demonstrated in the nucleus, thereby activating downstream transcriptional targets, many of which are involved in anti-inflammatory responses, cellular differentiation, and apoptosis [19, 20]. In addition to the activation of PPARγ, L-FABP has also been shown to be a downstream transcriptional target of PPARγ, suggesting the presence of a feedback loop involving cellular proliferation and inflammation [15, 19, 21].

L-FABP has been demonstrated to be overexpressed in various types of cancer, including colon, liver, gastric, and lung cancer. L-FABP has been shown to be significantly upregulated in clear cell renal cell carcinoma through epithelial-mesenchymal transition (EMT) [22], and fatty acid synthase has been shown to mediate the EMT of breast cancer cells [23]. EMT is currently the favored explanation for the distant metastasis of epithelial cancers including breast cancer [24]. Furthermore, in hepatocellular carcinoma, the expression of L-FABP has been associated with the expression of vascular endothelial growth factor (VEGF) [23, 25]. VEGF has been shown to be involved in the progression and prognosis of breast cancer, and it has been used to identify breast cancer patients at an increased risk of distant metastasis and recurrence [26]. However, the role of L-FABP in breast cancer is still poorly understood. Therefore, to address this issue, we conducted this study to investigate the association between plasma concentrations of L-FABP in patients with breast cancer and its expression in breast cancer tissue. We also explored the association between plasma L-FABP level and pretreatment hematological profile.

## Materials and methods

### Study participants

We enrolled 196 female patients with newly diagnosed breast cancer who underwent surgery at E-Da Hospital between January 2020 and July 2021. We also enrolled 57 age-matched women with normal mammography findings and no previous history of cancer who attended annual health examinations at E-Da Hospital as age-matched controls. All participants were asked to complete questionnaires on medical history, lifestyle behavior, family history of breast cancer and other cancers, menopause status, and reproductive and menstrual history. The participants completed the questionnaires before undergoing radio/chemotherapy and surgery, thereby minimizing the influence of treatment. All of the participants were informed of the study aims in detail, and they all provided written informed consent to participate. The Human Research Ethics Committee at E-Da Hospital approved this study.

### Anthropometric measurements and blood tests

All participants were of Han Chinese ethnicity and resided in the same area. They all underwent physical examinations and blood biochemical analyses after overnight fasting. Body weight was measured using a portable balance scale at an accuracy of 0.1 kg, and body mass index (BMI) was calculated as kg/m^2^. Seated blood pressure was also measured by a trained nurse with a digital automated blood pressure monitor (HEM-907, Omron, Japan) after a 5-min rest. Plasma albumin, alanine transaminase (ALT), aspartate transaminase (AST), glucose, creatinine, triglycerides, high-density lipoprotein cholesterol (HDL-C), low-density lipoprotein cholesterol (LDL-C), and total cholesterol were measured using a parallel multichannel analyzer (Hitachi 7170A, Tokyo, Japan) as reported previously [27, 28]. An automated cell counter (XE-2100 Hematology Alpha Transportation System, Sysmex Corporation, Kobe, Japan) was used for peripheral complete blood cell count. To rule out the presence of chronic infection and minimize confounding effects, participants with a white blood cell (WBC) count > 10.0 × 10^9^/l or < 4.0 × 10^9^/l were re-examined. Enzyme-linked immunosorbent assay (ELISA) (Cloud-Clone Corp., Katy, USA) was used to measure the concentrations of plasma L-FABP according to the manufacturer’s instructions. The analytical sensitivity was 0.59 ng/mL for L-FABP, and the specificity for human L-FABP was excellent. No significant interference or cross-reactivity with analogs was observed. All samples were measured twice in one experiment.

The fibrosis-4 index was calculated according to the formula reported by Vallet-Pichard et al. [29]: age (years) × AST (IU/l)/platelet count (10^9^/l)/√ALT (IU/l). The aspartate aminotransferase to platelet ratio index (APRI) was calculated as [(AST/Ul)/platelet count (× 10^3^)] × 100. The estimated glomerular filtration rate (eGFR) was calculated using the Chronic Kidney Disease Epidemiology Collaboration (CKD-EPI) two-concentration race equation [30]. All of the participants underwent eGFR measurement after 3 months of follow-up to confirm renal function status.

### Clinicopathologic characteristics of the tumors

Breast cancer was confirmed histologically, and progesterone receptor (PR) and estrogen receptor (ER) status were assessed. Breast cancer was staged according to the TNM system. The patients were classified according to tumor size (> 1 cm or ≤ 1 cm) and lymph node metastasis (N0+N1 or N2+N3). The histological grading of breast cancer that was based on the Bloom-Richardson system was used to determine the histological grade of breast cancer.

### Tissue samples collection

Due to the limited obtaining of permission for tissues for the investigation, not all patients signed informed consent. Hence, in the present study, samples from 42 consecutive consenting patients with newly diagnosed breast cancer who were surgically treated were collected from 2020 to 2021 at the General Surgery of E-Da Hospital. Samples of both cancerous and adjacent noncancerous breast tissue were obtained from these patients, none of whom had undergone chemotherapy or radiotherapy before surgery. All surgical specimens were fixed in 10% buffered formalin embedded in paraffin, and 4-μm-thick sections were cut for immunohistochemical (IHC) analysis and staining with hematoxylin and eosin. IHC staining was used to examine PR and ER status, and the standard HercepTest procedure (Dako 5204) was used for HER2/neu oncoprotein staining.

### Immunohistochemistry

For IHC staining, the following are the procedures: (a) deparaffinize sections, 3 changes of xylene, 10 min each; (b) re-hydrate in 2 changes of absolute alcohol, 5 min each; (c) 95% alcohol for 2 min; (d) 85% alcohol for 2 min; (e) 75% alcohol for 2 min; (f) wash 2 times in PBS buffer; (g) Hydrogen Peroxide Block to cover the sections for 10 min (Epredia, TL-125-QHD); (h) heat-mediated antigen retrieval: Tris-EDTA (pH9.0), 15 min; (i) Immunoblock, 5 min. (Epredia, TL-125-QHD); (j) primary antibody: L-FABP 1:500, 37 °C 1 h, wash 2 times in PBS buffer; (k) secondary antibody (Epredia, TL-125-QHD); (l) add 30 μl (1 drop) DAB Chromogen to 1.0 ml of DAB Buffer, mix by swirling and apply to tissue, 10 min (Epredia, TL-125-QHD); (m) counterstain in hematoxylin solution for 1 min, wash in running tap water 5 min; (n) dehydrate through 95% alcohol, 2 changes of absolute alcohol, 5 min each; (o) clear in 2 changes of xylene, 5 min each; and (p) mount with xylene-based mounting medium and then examined by light microscopy.

### Evaluation of immunohistochemical staining

The L-FABP staining results were scored according to the percentage of positively stained cells in 4 quantitative categories from score 1 to score 4 as < 25%, 25–50%, 51–75%, and > 75% positive cells, respectively. Two independent experts scored the staining separately for each specimen simultaneously under the same conditions. Any cases of discordant scores were rechecked and scored through consensus.

### Statistical analysis

The Kolmogorov-Smirnov test was used to check data normality. Normally distributed continuous variables were presented as mean ± SD, and nonnormally distributed variables were presented as median (interquartile range [IQR]). The unpaired Student’s *t*-test was used to analyze the differences in continuous variables. One-way analysis of variance was used to assess the effects of L-FABP among the tumor stage groups. As the distributions of ALT, APRI, monocyte count, serum triglycerides, and plasma L-FABP were skewed, the values were logarithmically transformed before analysis. Categorical variables were presented as frequency (percentage), and differences were analyzed using the chi-square test. Multiple logistic regression analysis was used to identify independent associations between the variables and the presence of breast cancer with the controls as a reference. Spearman rank correlation analysis was used to assess the associations among plasma L-FABP level and the other variables. A two-sided *p* value < 0.05 was considered to be statistically significant. All analyses were performed using the SAS statistical software, version 8.2 (SAS Institute Inc., Cary, NC, USA).

## Results

### Biochemical and clinical characteristics of the participants

The baseline biochemical and clinical data of the participants are shown in Table 1. Compared to the healthy controls, the plasma levels of L-FABP were significantly higher among breast cancer patients (7.6 ng/ml (IQR: 5.2–12.1) versus 6.3 ng/ml (IQR: 5.3–8.5, *p* = 0.008)). In addition, the patients had higher fasting glucose, BMI, systolic blood pressure (SBP), triglycerides, WBC count, monocyte count, neutrophil count, and lymphocyte count than the controls. The patients with breast cancer also had lower levels of hemoglobin than the controls. The mean age, total cholesterol, diastolic blood pressure, HDL-C, LDL-C, AST, ALT, AST:ALT ratio, APRI, fibrosis-4 index, creatinine, eGFR, albumin, hematocrit, red blood cell, and platelet counts were similar in the two groups.

**Table 1:** Baseline clinical and biochemical characteristics of the study population

Parameter	Breast cancer (*n* = 196)	Healthy controls (*n* = 57)	*p*-value
**Clinical data**			
Age (years)	55.4 ± 11.4	55.9 ± 8.8	0.795
Body mass index (kg/m^2^)	25.6 ± 4.4	22.1 ± 2.9	< 0.0001
SBP (mmHg)	130 ± 19	117 ± 17	< 0.0001
DBP (mmHg)	77 ± 11	74 ± 13	0.194
**Biochemical data**			
Fasting glucose (mg/dl)	121.3 ± 49.8	91.0 ± 6.3	< 0.0001
Total cholesterol (mg/dl)	195.8 ± 41.6	204.3 ± 33.4	0.198
Triglycerides (mg/dl)	127.0 (73.8–192.0)	79.0 (52.5–100.5)	0.0001
HDL-C	62.1 ± 15.4	63.3 ± 13.0	0.651
LDL-C	110.2 ± 37.8	107.5 ± 25.4	0.653
AST (U/l)	28.1 ± 17.7	24.5 ± 9.3	0.140
ALT (U/l)	19.0 (15.0–25.3)	17.0 (14.5–29.0)	0.383
AST:ALT ratio	1.3 ± 0.4	1.2 ± 0.4	0.783
APRI	0.1 (0.1–0.1)	0.1 (0.1–0.1)	0.272
Fibrosis-4 index	1.2 ± 0.8	1.1 ± 0.4	0.137
Creatinine (mg/dl)	0.90 ± 0.10	0.87 ± 0.20	0.244
Estimated GFR (ml/min/1.73 m^2^)	103.4 ± 16.4	107.6 ± 34.8	0.386
Albumin (g/dl)	4.3 ± 0.3	4.4 ± 0.2	0.480
WBC count (10^9^/l)	6.921 ± 2.414	4.982 ± 0.994	< 0.0001
Neutrophil count (10^9^/l)	4578 ± 2232	2959 ± 812	< 0.0001
Monocyte count (10^9^/l)	350 (279–474)	242 (212–272)	< 0.0001
Lymphocyte count (10^9^/l)	1838 ± 640	1632 ± 402	0.023
Red blood cells (× 10^6^/μl)	4.46 ± 0.49	4.57 ± 0.39	0.153
Hemoglobin (g/dl)	12.8 ± 1.5	13.2 ± 0.9	0.033
Hematocrit (%)	38.7 ± 4.6	39.7 ± 2.4	0.107
Platelet count (× 10^3^/μl)	269.4 ± 80.3	252.8 ± 56.2	0.146
L-FABP (ng/ml)	7.6 (5.2–12.1)	6.3 (5.3–8.5)	0.008

Data are mean ± SD or median (interquartile range)

*SBP* systolic blood pressure, *DBP* diastolic blood pressure, *HDL-C* high-density lipoprotein cholesterol, *LDL-C* low-density lipoprotein cholesterol, *AST* aspartate transaminase, *ALT* alanine transaminase, *APRI* AST to platelet ratio index, *GFR* glomerular filtration rate, *WBC* white blood cell count, *L-FABP* liver-type fatty acid-binding protein

### Associations between plasma L-FABP and breast cancer

Logistic regression analysis showed that plasma L-FABP concentrations were significantly associated with the presence of breast cancer (odds ratio 1.16, 95% confidence interval 1.05-1.27, *p* = 0.002) (Table 2). Even after controlling for confounding factors, including BMI, SBP, triglycerides, fasting glucose, AST, and ALT, L-FABP levels remained an independent risk factor for breast cancer (odds ratio 1.18, 95% confidence interval 1.04–1.32, *p* = 0.008).

**Table 2:** Logistic regression analysis of the association between liver-type fatty acid-binding protein and breast cancer

Variable	Odds ratio	95% confidence interval	*p*-value
Model 1			
L-FABP	1.16	1.05–1.27	0.002
Model 2			
L-FABP	1.09	1.00–1.20	0.048
Body mass index	1.23	1.10–1.39	< 0.0001
Systolic blood pressure	1.02	1.00–1.05	0.034
Model 3			
L-FABP	1.14	1.02–1.28	0.020
Body mass index	1.20	0.99–1.45	0.057
Systolic blood pressure	0.99	0.96–1.03	0.751
Triglycerides	1.01	0.99–1.02	0.113
Fasting glucose	1.05	0.99–1.10	0.059
Model 4			
L-FABP	1.18	1.04–1.32	0.008
Body mass index	1.26	1.03–1.55	0.027
Systolic blood pressure	1.00	0.96–1.03	0.823
Triglycerides	1.01	0.99–1.02	0.171
Fasting glucose	1.06	1.00–1.12	0.035
Aspartate transaminase	1.04	0.97–1.11	0.279
Alanine transaminase	0.96	0.92–1.00	0.055

*L-FABP* liver-type fatty acid-binding protein

### Plasma L-FABP levels and clinicopathological features of the tumors

**Fig. 1:**
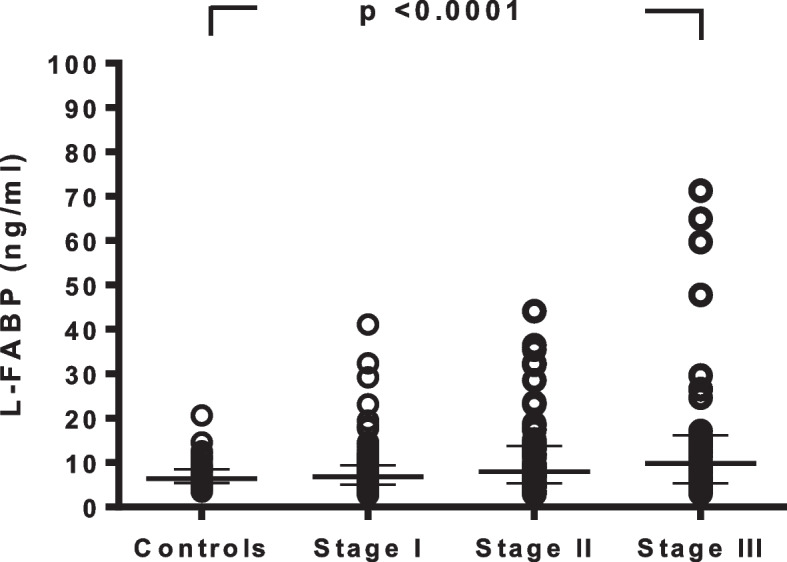
Associations between liver-type fatty acid-binding protein and stage progression of breast cancer. Bars represent the median (interquartile range). Differences between the groups were analyzed using one-way analysis of variance

The patients were classified into two groups according to the median L-FABP level (7.6 ng/mL), and the relationships between the clinicopathological features of the tumors and plasma L-FABP levels were analyzed (Table 3). There were no significant differences in tumor size, lymph node metastasis, histologic grade, or PR status. The pathologic stage T2+T3+T4 (*p* = 0.045), clinical stage III (p = 0.045), negative ER status (*p* = 0.031), and positive HER-2 receptor status (*p* = 0.029) were significantly higher in the patients with an L-FABP level greater than the median. Linear contrast analysis showed that L-FABP levels gradually increased as the stage increased (*p* < 0.0001) (Fig. 1). However, when we have calculated plasma L-FABP levels among patients stratified by the four types of breast cancer (ER+/PR+/HER2− vs. ER+/PR−/HER2+ vs. ER−/PR−/HER2+ vs. ER−/PR−/HER2−), the median plasma L-FABP levels of breast cancer patients with different types of breast cancer (ER+/PR+/HER2− vs. ER+/PR−/HER2+ vs. ER−/PR−/HER2+ vs. ER−/PR−/HER2−) did not show any difference among the groups (9.0 ng/ml [interquartile range 6.2 to 13.6] vs. 8.6 ng/ml [interquartile range 6.0 to 12.8] vs. 6.9 ng/ml [interquartile range 5.2 to 13.5] vs. 4.4 ng/ml [interquartile range 4.1 to 8.1], *p* = 0.636) (data not shown). Please note that the number of patients is too small to draw any conclusion.

**Table 3:** Associations between plasma liver-type fatty acid-binding protein and clinicopathological characteristics in the breast cancer patients

Parameters	L-FABP (ng/ml)		*p*-value
	> 7.6 (*n* = 98)	≤ 7.6 (*n* = 98)	
Tumor size (cm)			
≤ 1	25 (25.5)	37 (37.8)	0.065
> 1	73 (74.5)	61 (62.2)	
Pathologic T stage			
T0+T1	39 (39.8)	53 (54.1)	0.045
T2+T3+T4	59 (60.2)	45 (45.9)	
Lymph node metastasis			
N0+N1	84 (85.7)	92 (93.9)	0.059
N2+N3	14 (14.3)	6 (6.1)	
Histologic grade			
1	51 (52.0)	58 (59.2)	0.314
≥ 2	47 (48.0)	40 (40.8)	
Clinical stage			
Stage I	36 (36.7)	53 (54.1)	0.015
Stage II	38 (38.8)	31 (31.6)	0.295
Stage III	24 (24.5)	14 (14.3)	0.045
Estrogen receptor status			
Positive	77 (78.6)	88 (89.8)	0.031
Negative	21 (21.4)	10 (10.2)	
Progesterone receptor status			
Positive	74 (75.5)	66 (67.4)	0.206
Negative	24 (24.5)	32 (32.7)	
HER-2 receptor status			
Positive	62 (63.3)	47 (48.0)	0.029
Negative	36 (36.7)	51 (52.0)	

Data are number (%)

### Correlations among L-FABP level and pretreatment hematologic parameters in breast cancer patients and controls

L-FABP was significantly positively associated with age, BMI, SBP, DBP, fasting glucose, AST, ALT, AST:ALT ratio, APRI, fibrosis-4 index, creatinine, monocyte count, and lymphocyte count and negatively associated with eGFR in the breast cancer group (Table 4). In addition, L-FABP was significantly positively correlated with age, BMI, SBP, DBP, fasting glucose, triglycerides, AST, ALT, AST:ALT ratio, APRI, fibrosis-4 index, and creatinine and negatively associated with eGFR in the control group (Table 4).

**Table 4:** Spearman correlation analysis of clinical and biochemical variables with plasma levels of liver-type fatty acid-binding protein

Parameter	Breast cancer (*n* = 196)	*p*-value	Healthy controls (*n* = 57)	*p*-value
Age	0.320	< 0.0001	0.537	< 0.0001
Body mass index	0.353	< 0.0001	0.273	0.040
SBP	0.288	< 0.0001	0.268	0.044
DBP	0.158	0.027	0.281	0.034
Fasting glucose	0.282	0.018	0.448	0.001
Total cholesterol	− 0.176	0.095	0.091	0.500
Triglycerides (mg/dl)	0.180	0.094	0.278	0.036
HDL-C	− 0.168	0.122	− 0.089	0.513
LDL-C	− 0.183	0.088	0.059	0.673
AST	0.352	< 0.0001	0.526	< 0.0001
ALT	0.443	< 0.0001	0.515	< 0.0001
AST:ALT ratio	0.369	< 0.0001	0.297	0.025
APRI	0.191	0.012	0.380	0.004
Fibrosis-4 index	0.173	0.018	0.318	0.016
Creatinine	0.315	< 0.0001	0.342	0.009
Estimated GFR	− 0.386	< 0.0001	− 0.477	0.0002
Albumin	0.108	0.314	0.059	0.668
WBC count	0.126	0.079	− 0.002	0.988
Neutrophil count	0.122	0.119	− 0.069	0.612
Monocyte count	0.263	0.001	− 0.148	0.273
Lymphocyte count	0.161	0.038	0.121	0.371
Red blood cells	0.103	0.153	0.033	0.806
Hemoglobin	0.084	0.244	0.242	0.069
Hematocrit	0.019	0.794	0.185	0.169
Platelet count	0.020	0.785	0.016	0.905

*SBP* systolic blood pressure, *DBP* diastolic blood pressure, *HDL-C* high-density lipoprotein cholesterol, *LDL-C* low-density lipoprotein cholesterol, *AST* aspartate transaminase, *ALT* alanine transaminase, *APRI* AST to platelet ratio index, *GFR* glomerular filtration rate, *WBC* white blood cell count

### L-FABP immunohistochemical data and TNM stage of the patients

**Fig. 2:**
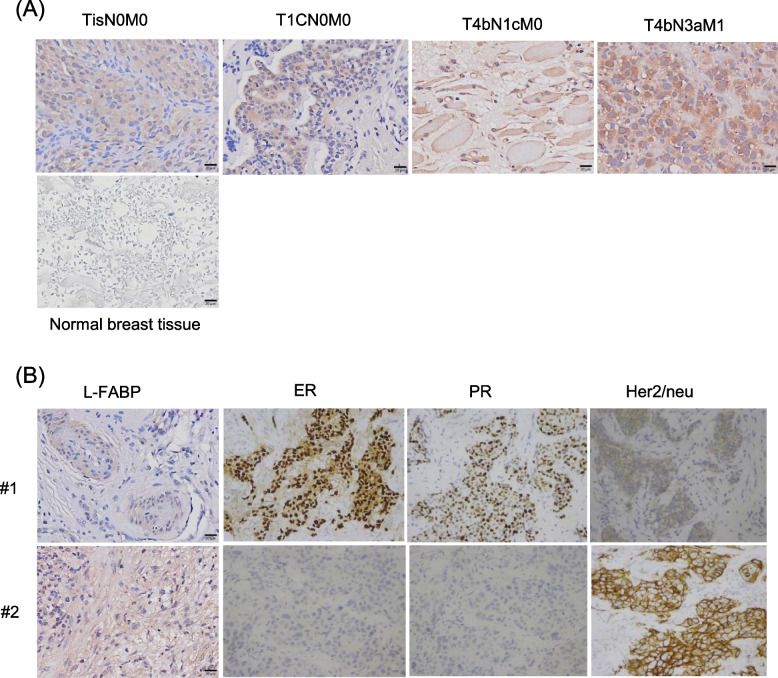
The expression of the liver-type fatty acid-binding protein (L-FABP) in breast cancer tissues. The expression of L-FABP in breast cancer tissues, as determined by immunohistochemistry, was according to TNM state. The expression of L-FABP was detected in the cytoplasmic, nuclear, or both cytoplasmic and nuclear of all breast cancer tissue examined, not in the normal tissue (**A**). Representative immunohistochemistry images for the localization of L-FABP, ER, PR, and Her2/neu in 2 cancer tissues (#1 and #2) (**B**)

We further investigated the levels of L-FABP in breast cancer tissues using IHC analysis. The detailed L-FABP immunohistochemical data and TNM state of these 42 patients are shown in Additional file 1: Table S1. Furthermore, the expression of L-FABP in breast cancer tissues was according to TNM state. The expression of L-FABP was detected in the cytoplasm, nuclear, or both cytoplasm and nuclear of all breast cancer tissue examined, not in the normal tissue (Fig. 2A). Moreover, the IHC results for the localization of L-FABP, Her2/neu, PR, and ER in cancer tissues showed that the expression of L-FABP was negatively correlated with PR and ER expressions and positively correlated with the expression of Her2/neu (Fig. 2B).

## Discussion

To the best of our knowledge, this is the first study to evaluate the concentration and expression of L-FABP in breast cancer patients. Our results showed significantly higher plasma L-FABP concentrations in the patients with breast cancer than in the controls, and also a significantly elevated L-FABP expression in breast cancer tissue. Furthermore, the rates of pathologic stage T2+T3+T4, clinical stage III, positive HER-2 receptor status, and negative ER status were significantly higher in the patients with an L-FABP level greater than the median. Moreover, we found a gradual increase in the concentration of L-FABP with an increase in the stage. This is in agreement with previous reports that epidermis-, heart-, and L-FABP may play a key role in the progression of invasiveness and metastasis in human breast cancer [31]. In addition, the authors concluded that the secretion of these FABPs has the potential to serve as a diagnostic marker of breast cancer. In their study, they focused on FABP expressions in 35 patients with ductal infiltrating carcinoma and 16 with fibroadenoma of the breast; however, we extended this to the association between concentration and expression of L-FABP and breast cancer. Moreover, Li et al. found that epidermis-, heart-, and L-FABP expressions were significantly upregulated in ductal infiltrating carcinoma compared with benign tissue [31]. Our results provide a new viewpoint to previous studies, as we found that the association between plasma L-FABP level and breast cancer was independent of BMI. In 1995, Woodford et al. demonstrated the interactions between the cell membrane and L-FABP [32]. However, since then, the majority of studies have concentrated on its role in regulating lipid metabolism and transporting fatty acids [33]. Moreover, the co-expression of VEGF with L-FABP has been reported in the cell membrane [23], and VEGF expression has been reported to be a prognostic factor for invasive breast cancer [34] and to promote the proliferation of other cell types, including breast tumor cells [35]. Our results of an association between L-FABP concentration and expression with breast cancer and a gradual increase in concentration with increasing stage are consistent with previous studies [34, 35] and suggest the possibility of a link between L-FABP and cell proliferation and fatty acid and lipid metabolism responses. This may be a mechanism for the progression of breast cancer.

The biological mechanisms underlying the role of L-FABP in breast cancer pathogenesis have yet to be clarified. Chronic inflammation has been demonstrated in tumors, and this may be associated with chemoresistance and cancer progression. L-FABP is an intracellular protein responsible for the transportation of long-chain fatty acids. In addition to its functions in lipid metabolism and cellular differentiation, FABP1 also plays a role in inflammation through interactions with PPARs [16]. Furthermore, PPARs have been shown to regulate inflammation. Of note, PPARγ has been shown to be involved in macrophage and monocyte differentiation. Since L-FABP is a known transactivator of PPARγ, the simultaneous expression of both L-FABP and PPARγ may have consequences with regard to the PPARγ activation in alveolar macrophages [36]. Moreover, a previous study showed the biological activity of human L-FABP by demonstrating that its human recombinant form induces interleukin (IL)-6 production in whole blood cells and human cell lines [37]. In addition, the similar effect of L-FABP and IL-1a on whole blood cells indicates that the circulating or extracellular form of L-FABP may be a mediator of systemic inflammation [37]. We found higher plasma L-FABP levels in the patient group in this study, and the higher plasma L-FABP levels were associated with monocyte count and lymphocyte count only in breast cancer patients. Taken together, we suggest that L-FABP may be a marker of inflammation that participates in the process of breast cancer.

In the current study, we evaluated the correlations between plasma L-FABP levels and liver damage enzymes and liver fibrosis score including ALT, AST, AST:ALT ratio, APRI, and fibrosis-4 index. L-FABP is widely expressed in hepatocytes and is known to play a major role in promoting cell proliferation [38], and it is one of the factors responsible for hepatic regeneration [39]. A previous report demonstrated that regenerating livers could induce acute-phase responses and increased expressions of acute-phase cytokines [40], which could in turn play a role in the development of breast cancer [41]. Furthermore, a previous study provided evidence that increased serum L-FABP levels indicated ongoing liver damage in patients with NAFLD and showed relationships between L-FABP and BMI, glucose, AST, ALT, and γ-glutamyltransferase. Thus, L-FABP may be an independent predictor of NAFLD [42]. Moreover, previous studies have reported a significant association between NAFLD and breast cancer [7, 10, 43]. The mechanisms underlying extrahepatic carcinogenesis in a fatty liver are not completely understood. Muhidin et al. [44] reported three major factors that may explain the mechanistic link. The first factor is through high levels of inflammatory cytokines, especially tumor necrosis factor-alpha. These inflammatory cytokines have been shown to promote increases in circulating triglycerides, insulin resistance, growth, apoptosis, and tumor cell proliferation in many cancers [45]. The second factor is high levels of leptin and hyperinsulinemia, which have been shown to induce carcinogenesis [46]. By binding to circulating sex hormone-binding globulin, elevated insulin levels lead to increased secretion of estrogen. This increase in estrogen then mediates downstream signaling, potentially leading to breast carcinogenesis [47]. The third factor is a decrease in adiponectin levels, which can lead to marked insulin resistance and a subsequent increase in insulin growth factor-1 (IGF-1) levels. Insulin binds to IGF-1 receptors and plays an important role in apoptosis, cell proliferation, and increased production of VEGF. Further studies are needed to assess this association and explore the mechanistic link between fatty liver infiltration and breast cancer.

L-FABP has high specificity for binding to hydrophobic lipid ligands, and it is widely expressed in the cytoplasm. L-FABP overexpression has been observed in different types of cancer; however, its role in breast cancer remains unclear. In the present study, we observed high L-FABP staining in breast cancer tissue. However, a limitation of this study is that the number of tumors is too small to draw any definite conclusions (Additional file 1: Table S1). Further studies are needed to verify the importance of the protein expression of L-FABP in these tumors. Moreover, a previous study showed nuclear and cytoplasmic staining for L-FABP in colorectal carcinomas [48]. In the present study, we demonstrated cytoplasmic, nuclear, or both cytoplasmic and nuclear staining for L-FABP, which is consistent with these findings, thereby raising the possibility L-FABP may play a role in breast cancer.

Carlsson et al. reported that growth hormone is an important regulator of L-FABP metabolism in vivo and in vitro [49]. Interestingly, in our study, an L-FABP level greater than the median in patients with negative ER status and positive HER-2 receptor status and the expression of L-FABP was negatively correlated with the expression of ER and PR and was positively correlated with the expression of HER-2. Taken together, our results clearly showed that L-FABP is a promising and novel prognostic factor for breast cancer. There are several limitations to this study. First, the number of patients was relatively small, and future studies are needed with a larger sample size. Second, the cross-sectional design limited the inference of causal relationships between L-FABP and breast cancer. Prospective cohort studies are needed to elucidate the role of L-FABP as a biomarker of breast cancer and the causative association between breast cancer and changes in L-FABP level. Third, although we controlled for other major cancer risk factors, we cannot rule out the possibility of unmeasured confounding factors. Finally, we lack information and molecular mechanism regarding the function of L-FABP in breast cancer pathogenesis in this report. Analysis using well-known published online cancer genome databank such as The Cancer Genome Atlas and Gene Expression Omnibus or in vitro study using well-established breast cancer cells lines, such as MCF-7, MDA-MB-231, SkBr3, and T-47D is warranted.

## Conclusion

In conclusion, the present study demonstrated that L-FABP expression and concentration are higher in breast cancer, suggesting that L-FABP may play a role in the pathogenesis of breast cancer. Further studies are needed to investigate the precise mechanisms by which L-FABP signaling is involved in the development of breast cancer and establish new therapeutic strategies and diagnostics using L-FABP as the target.

## Supplementary Information

**Additional file 1: Table S1.** Patients’ liver type-fatty acid-binding protein immunohistochemical data and TNM state.

## Data Availability

The datasets used and/or analyzed during the current study are available from the corresponding author upon reasonable request.

## Abbreviations

NAFLD: Nonalcoholic fatty liver disease
FABP: Fatty acid-binding protein
PPARs: Peroxisome proliferator-activated receptors
L-FABP: Liver type-fatty acid-binding protein
EMT: Epithelial-mesenchymal transition
VEGF: Vascular endothelial growth factor
BMI: Body mass index
ALT: Alanine transaminase
AST: Aspartate transaminase
HDL-C: High-density lipoprotein cholesterol
LDL-C: Low-density lipoprotein cholesterol
WBC: White blood cell
ELISA: Enzyme-linked immunosorbent assay
APRI: Aspartate aminotransferase to platelet ratio index
eGFR: Estimated glomerular filtration rate
CKD-EPI: Chronic Kidney Disease Epidemiology Collaboration
PR: Progesterone receptor
ER: Estrogen receptor
IHC: Immunohistochemical
IQR: Interquartile range
SBP: Systolic blood pressure
IL: Interleukin
IGF-1: Insulin growth factor-1
