# Supplementary material for: Plasma levels and tissue expression of liver-type fatty acid-binding protein in patients with breast cancer
Source: World J Surg Oncol. 2023 Feb 18;21:52. doi: 10.1186/s12957-023-02944-8 (PMC9938596; doi:10.1186/s12957-023-02944-8)
Supplement: Supplementary file 1 — Additional file 1: Table S1. Patients’ liver type-fatty acid-binding protein immunohistochemical data and TNM state. [file 12957_2023_2944_MOESM1_ESM.docx]

Table S1. Patients’ liver type-fatty acid-binding protein immunohistochemical data and TNM state

| Case no. | IHC score | Positive tumor cell% | Staining intensity | Staining position | TNM state |  |
| --- | --- | --- | --- | --- | --- | --- |
| 1 | 1 | 8.2 | + | c | T2N1aM0 | |
| 2 | 2 | 48.7 | + | c + n | T2N1aM0 |  |
| 3 | 2 | 30 | + | c | T1cN0M0 |  |
| 4 | 3 | 62 | + | c + n | T4N3M1 |  |
| 5 | 3 | 60.4 | + | c + n | T4N1M1 |  |
| 6 | 3 | 70.8 | + | c | T1bN0M0 |  |
| 7 | 3 | 61 | + | c | TisN0M0 |  |
| 8 | 3 | 53.9 | ++ | c + n | T2N0M0 |  |
| 9 | 3 | 60.4 | + | c + n | T2N0M0 |  |
| 10 | 3 | 67.2 | + | c + n | T2N0M0 |  |
| 11 | 3 | 75 | + | c + n | T2N0M0 |  |
| 12 | 3 | 70.8 | + | c | T2N0M0 |  |
| 13 | 3 | 64.9 | + | c + n | T1cN0M0 |  |
| 14 | 3 | 61.6 | + | c + n | T2N0M0 |  |
| 15 | 4 | 87.5 | ++ | c | T2N1aM0 |  |
| 16 | 4 | 97 | +++ | c + n | T1cN1aM0 |  |
| 17 | 4 | 89.2 | ++ | c + n | T0N0M0 |  |
| 18 | 4 | 98 | +++ | c + n | T4N1M1 |  |
| 19 | 4 | 81.8 | + | c | T2N0M0 |  |
| 20 | 4 | 80.4 | ++ | c + n | T2N0M0 |  |
| 21 | 4 | 84.1 | + | c | T2N0M0 |  |
| 22 | 4 | 85 | +++ | c + n | T3N0M0 |  |
| 23 | 4 | 75.9 | + | c + n | T2N3aM0 |  |
| 24 | 4 | 93.4 | +++ | c + n | T4bN3aM1 |  |
| 25 | 4 | 95 | +++ | c + n | T4bN0M0 |  |
| 26 | 4 | 91.3 | ++ | c + n | T1cN0M0 |  |
| 27 | 4 | 86.4 | +++ | c + n | T1cN1aM0 |  |
| 28 | 4 | 97 | +++ | c + n | T1cN0M0 |  |
| 29 | 4 | 89.9 | +++ | c + n | T1bN0M0 |  |
| 30 | 4 | 96 | +++ | c + n | T1bN0M0 |  |
| 31 | 4 | 79.4 | +++ | c + n | T1bN0M0 |  |
| 32 | 4 | 88.7 | +++ | c + n | T1aN0M0 |  |
| 33 | 4 | 83.6 | ++ | c + n | T1cN0M0 |  |
| 34 | 4 | 95 | +++ | c + n | T1cN0M0 |  |
| 35 | 4 | 87.9 | ++ | c + n | T2N0M0 |  |

Table S1. Patients’ liver type-fatty acid-binding protein immunohistochemical data and TNM state

| Case no. | IHC score | Positive tumor cell% | Staining intensity | Staining position | TNM state |
| --- | --- | --- | --- | --- | --- |
| 36 | 4 | 93.9 | +++ | c + n | T4bN1cM0 |
| 37 | 4 | 88 | +++ | c + n | T1bN0M0 |
| 38 | 4 | 92.4 | ++ | c + n | T3N2aM0 |
| 39 | 4 | 90 | +++ | c + n | T1cN0M0 |
| 40 | 4 | 94.5 | +++ | c + n | T1cN0M0 |
| 41 | 4 | 90.9 | +++ | c + n | T2N0M0 |
| 42 | 4 | 86 | +++ | c + n | T1cN0M0 |

Immunohistochemical score: 1:<25%, 2: 25-50%, 3: 50-75%, 4: >75%. c, cytoplasmic; n, nuclear; IHC, immunohistochemical.
